# Spillover: Mechanisms, Genetic Barriers, and the Role of Reservoirs in Emerging Pathogens

**DOI:** 10.3390/microorganisms12112191

**Published:** 2024-10-30

**Authors:** Silvia Pauciullo, Verdiana Zulian, Simone La Frazia, Paola Paci, Anna Rosa Garbuglia

**Affiliations:** 1 Laboratory of Virology, National Institute for Infectious Diseases “Lazzaro Spallanzani” (IRCCS), 00149 Rome, Italy; silvia.pauciullo@inmi.it (S.P.); verdiana.zulian@inmi.it (V.Z.); 2 Department of Biology, University of Rome Tor Vergata, Via della Ricerca Scientifica 1, 00133 Rome, Italy; simone.la.frazia@uniroma2.it; 3 Department of Computer, Control, and Management Engineering “A. Ruberti” (DIAG), Sapienza University of Rome, 00185 Rome, Italy; paci@diag.uniroma1.it

**Keywords:** spillover, zoonoses, viral evolution, epistasis, virus transmission

## Abstract

Viral spillover represents the transmission of pathogen viruses from one species to another that can give rise to an outbreak. It is a critical concept that has gained increasing attention, particularly after the SARS-CoV-2 pandemic. However, the term is often used inaccurately to describe events that do not meet the true definition of spillover. This review aims to clarify the proper use of the term and provides a detailed analysis of the mechanisms driving zoonotic spillover, with a focus on the genetic and environmental factors that enable viruses to adapt to new hosts. Key topics include viral genetic variability in reservoir species, biological barriers to cross-species transmission, and the factors that influence viral adaptation and spread in novel hosts. The review also examines the role of evolutionary processes such as mutation and epistasis, alongside ecological conditions that facilitate the emergence of new pathogens. Ultimately, it underscores the need for more accurate predictive models and improved surveillance to better anticipate and mitigate future spillover events.

## 1. Introduction

The term “spillover” has gained significant attention in scientific literature, particularly after the SARS-CoV-2 pandemic. Despite its widespread use, a clear and consistent explanation of the factors driving spillover events has yet to be established. Generally, “spillover” refers to the transmission of a pathogen from one species to another, including humans. However, it is often incorrectly used to describe the spread of a pathogen from a known reservoir to a pre-existing host, a situation that would be better described as the emergence or re-emergence of a pathogen. A recent study by Gavotte et al. (2023) identified ten different and contradictory definitions of spillover in the literature, none of which fully explain the phenomenon [1]. For instance, Morris (2011) defines spillover in the context of cholera, where the transmission occurs from an environmental reservoir (such as water) to humans [2]. In contrast, Alexander (2012) focuses on the jump of a pathogen from one animal species to another, independent of the transmission mode [3]. A key point common across these definitions is that the pathogen variant responsible for spillover must already be present in the reservoir species.

In 1995, Chinery defined virus spillover from a reservoir to humans as a rare event [4]. Later, in 2000, Daszak described spillover as “The transmission of infectious agents from reservoir animal populations (often domesticated species) to sympatric wildlife, termed spillover, underpins the emergence of a range of wildlife EIDs” [5]. In 2004, Power and Mitchell stated that a process known as the “spillover effect” or “pathogen spillover” occurs when “the pathogen reaches high prevalence in a reservoir and then spills over into another host” [6]. Other definitions introduce intermediate hosts, such as insects, that facilitate pathogen transmission between species. There are also more anthropocentric definitions focusing on pathogen transmission from animals to humans. Often, “spillover” is used with the meaning of “interspecies transmission”.

These different definitions reflect the complexity of the term “spillover”, which encompasses a wide range of transmission mechanisms and ecological contexts. For this reason, it is important to provide a clear definition of spillover to avoid causing alarm and confusion in the public in the event of outbreaks of emerging or re-emerging pathogens, which are often referred to as “spillover” by the media.

Various aspects of spillover have been defined, such as **spillover rate** (the number of spillover events within a specific host–parasite system) and **spillover diversity** (the number of parasite species involved in spillover events) [7]. The transmission of a new pathogen also involves environmental factors that may facilitate its spread, such as host species density, the presence of multiple species that can serve as new hosts, or climate changes that allow the introduction of new species into an ecosystem. Consequently, a positive relationship between biodiversity and spillover has been proposed [8,9,10,11]. It has also been suggested that **parasite diversity** increases with host diversity [12]. However, little is known about the specific factors that determine spillover [13].

In this review, after providing a general overview of zoonoses, which are the primary sources of spillover and emerging pathogens, we will examine the factors contributing to the emergence of new variants in reservoir species that are capable of “jumping” and adapting to a new host species. These factors include: (1) genetic variability of the virus in the reservoir species; (2) the concept of fitness, which allows a genome/strain/variant to successfully cross from one species to another (especially humans); (3) genetic barriers that either hinder (restriction factors, innate and cell-mediated immunity) or facilitate (receptors) viral entry into a new host; and (4) the adaptation and spread of the virus in the new host, as measured by R_0_ and R_e_.

## 2. General Aspects of Emerging Pathogens

Until the 1990s, it was widely believed that the mortality rate from infectious diseases was in decline due to advances in hygiene, the introduction of vaccines (e.g., hepatitis B virus vaccine), and the development of therapies and antibiotics. However, human mortality from infectious diseases has remained steady at around 13–15 million deaths per year, and this number tends to persist until at least 2030 [14]. Over the past two decades, outbreaks caused by level 4 pathogens, such as Ebola and Nipah, as well as global pandemics like SARS-CoV-2, have posed significant challenges to both national and international healthcare systems and also caused substantial economic losses [15,16]. The first major pandemic of the 20th century, known as the “Spanish” influenza (1918–1920), resulted from a zoonotic spillover of the H1N1 influenza virus, causing over 50 million deaths. Alongside World War I, this pandemic led to the collapse of healthcare systems due to hospital overcrowding and triggered a macroeconomic disaster, with a GDP decline of 6–8% in the affected nations [17]. Approximately a century later, the COVID-19 pandemic led to over 7 million deaths and precipitated the collapse of both global healthcare and economic systems. Many countries implemented full or partial lockdowns to control the viral spread, severely slowing the global economy, halting numerous industries, and resulting in widespread job losses [18]. The resilience demonstrated during the COVID-19 crisis has introduced new perspectives for emergency response, such as remote learning and teleworking. The latter remains in use in the post-pandemic phase as a means of reducing infrastructure costs and mitigating transportation congestion.

An analysis of emerging infectious disease (EID) events from 1940 to the present shows that the majority (60.3%) are zoonotic, with 71.8% originating from wildlife (e.g., SARS-CoV-2, Ebola virus), a trend that has increased over time [16]. Viruses and prions account for 25.4% of these EID events. Vector-borne pathogens are responsible for 22.8% of EIDs, with 30% of cases occurring over the past decade [16]. Among these, RNA viruses represent a particularly high risk for spillover due to their elevated mutation rates, non-homologous recombination, and genome reassortment in segmented viruses which promote adaptation to new host species. It is estimated that approximately 20% of known mammalian viruses are capable of jumping to humans. However, given that only 0.1% of mammalian-infecting viruses have been identified, the risk of cross-species transmission is substantial [19,20].

For example, a large number of SARS-related coronaviruses circulate among bats in China and Southeast Asia, and 2/3 of their reservoirs have yet to be identified [21,22]. Despite the high genetic diversity of viruses, most have a limited host range. Within the “virosphere”, 73.3% of viruses are associated with only one or two species, 3.5% with three to four species, and 22.5% with more than four species. The transmission network remains largely cryptic [23].

RNA viruses present the greatest threat to human health, with 214 species known to cause infections in humans. These viruses belong to 55 genera across 21 families. Among RNA viruses involved in zoonoses, the *Bunyaviridae* family is the most represented, with 40 species known to infect humans [24,25]. Other prominent families include *Filoviridae*, *Orthomyxoviridae*, and *Togaviridae* [26,27].

A viral reservoir host is a species in which a viral pathogen circulates endemically, establishing a co-evolutionary process with the host. A specific type of reservoir host is the intermediate host, which plays a role in viral natural selection and/or adaptation. There are no clearly defined characteristics that identify specific reservoirs that facilitate spillover. Even phylogenetic distance does not appear to be a determining factor. For instance, despite their evolutionary distance from humans, rodents and bats (Chiroptera) are major zoonotic reservoirs [28,29,30]. Bats, in particular, are known to harbour numerous viruses, including rabies, Hendra, Nipah, Ebola, SARS-CoV, MERS-CoV, and SARS-CoV-2. Their immune systems may manage viral infections through a balance between immune defense and tolerance, rather than through elevated body temperature and metabolism, as they are the only flying mammals [31,32,33,34,35]. Ecologically, their high species diversity and shared habitats with humans and livestock further enhance zoonotic risk [36]. Similarly, rodents, such as *Mastomys natalensis*, the primary reservoir for the Lassa virus, also exhibit zoonotic potential [37]. Both bats and rodents can carry viruses without displaying clinical symptoms, potentially through similar immune mechanisms [38]. However, some viruses, such as Lyssavirus, are abundant in bats but are not zoonotic, indicating that much remains to be understood about spillover mechanisms.

It is estimated that there are about 1.6 million viruses capable of infecting humans [19]. This estimated number, compared to the over 270 viral species currently known to infect human that pose zoonotic risks [27,39], suggests that 99.9% of zoonotic viruses remain undiscovered. A major limitation in identifying human viruses is that they are often only detected after causing disease in humans but, as observed for other pathogens [40], the presence of zoonotic orphan viruses cannot be excluded.

In recent years several projects aimed at identifying viruses through metagenomics have been initiated. The PREDICT project, for example, has identified more than 1000 new viruses, including SARS-like coronaviruses capable of infecting human cells [41]. The project operated across 35 countries for eight years [42]. Furthermore, the Global Virome Project seeks to identify the majority of unknown viral diversity, the ecological and environmental characteristics where viruses are identified, and collects metadata that may be useful for analysing the risk of viral spillover in humans (https://www.globalviromeproject.org/, accessed on 17 September 2024). The majority of non-human reservoirs are mammals, such as rodents, ungulates, and bats, while a smaller proportion of viruses infect birds. Rarely, reservoirs different from mammals and birds are identified.

It is important to differentiate spillover from human migration, which can introduce pathogens into new geographical areas. For instance, the colonization of the Americas led to the spread of smallpox, typhus, and measles among the native populations, causing an estimated 50 million deaths [5].

Various models have been developed to predict spillover events [27]. These models consider factors such as the number of zoonotic viruses and their potential to infect multiple species to create predictive frameworks for each zoonotic species [43]. However, the predictive accuracy of these models rarely exceeds 30%, highlighting the importance of ongoing surveillance of viral pathogens rather than relying solely on prediction models to inform public health decisions.

Much attention has been focused on post-spillover events, specifically, how viruses adapt to and spread within new host species. However, less attention has been paid to the initial steps leading to spillover and the factors that may trigger it.

## 3. Factors That Favor Spillover

In this section, we aim to outline the key factors that precede a species jump, particularly focusing on the emergence of a viral variant in the so-called “reservoir” species before it adapts to a new host. Spillover is not a simple passive event; if it were, such events would be much more common. We will give a description of how and why viruses mutate. For spillover to occur, the virus must mutate and develop adequate fitness within the reservoir species before it can be transmitted to a new host. These mutations often arise during extended periods of viral presence in the reservoir, highlighting that spillover is part of a continuous evolutionary process. The first adaptive step always occurs in the reservoir species before the virus can infect and propagate in a new species, leading to the spread of the pathogen.

The emergence of a new virus through spillover can be divided into three stages. First, the virus must acquire the ability to infect the cells of a new host species. Second, it must adapt to the new host to facilitate inter-host transmission within the new species. Third, the virus must develop the ability to spread within the host population, an ecological process that is sometimes aided by vectors.

The mutated pathogen will generate genetic variability that results in mutants capable of adapting to a new host. This is more likely in viruses with high replication rates and error-prone replication processes that introduce mutations into the genome. RNA viruses, in particular, are prone to spillover due to their high mutation rates. For instance, the mutation rate in RNA viruses is estimated to be between 0.1 and 1.0 mutations per genome per replication [44], a rate significantly higher than that of DNA viruses due to the lower fidelity of RNA-dependent RNA polymerases resulting from the lack of proofreading activity. The mutation rate in RNA viruses is estimated to be six times higher than that observed in eukaryotes [44,45]. This characteristic results in RNA viruses being key agents of emerging and re-emerging diseases.

The high mutation rate limits the size of the viral genome by establishing an error threshold that leads RNA viruses to evolve relatively small genomes, typically around 10 kb. Larger genomes would accumulate lethal mutations over time, compromising viral fitness and survival [46,47]. For example, the substitution rate of the Influenza A/H1N1 pandemic virus is estimated at approximately 5 × 10^−3^ substitutions/site/year [48]. Coronaviruses are an exception as they can have genomes around 30 kb due to the presence of proofreading mechanisms [49].

Because of their mutation-prone nature, viral populations often exist as “quasi-species”. This diversity can enhance the likelihood of spillover by facilitating the emergence of variants capable of interacting with receptors in a new host species [50,51,52]. However, many mutations are lethal or reduce fitness; for instance, studies on RNA viruses suggest that 40–80% of mutations are lethal [53]. On the other hand, if a genome did not mutate at all this would lead to the disappearance of the species because its ability to adapt to environmental variations would be compromised. In some cases, as for the vesicular stomatitis virus (VSV), it has been estimated that 40% of mutations are lethal for the host cell, while in the case of poliovirus, only about 1% of virions released by a single cell are capable of completing the replication cycle [54].

Interestingly, many mutations that aid adaptation to a new host are likely deleterious in the reservoir host [55]. This suggests that RNA viruses may not be as adaptable as previously thought, with many cross-species transmissions resulting in transient spillovers rather than long-term establishment in the new host, as seen with the West Nile virus or avian influenza H5N1 in humans [56]. Moreover, mutation rates vary across viral genes and are also influenced by whether or not an intermediate host is needed. For example, vector-borne RNA viruses exhibit lower mutation rates (d_N_/d_S_ ratio; d_N_, nonsynonymous substitutions; d_S_, synonymous substitutions) in structural protein genes compared to those transmitted through other routes: d_N_/d_S_ of 0.066 and 0.165, respectively (*p* = 0.018, Mann–Whitney U test) [57].

When adaptation to a new host does not occur, spillover events result in only sporadic infections in humans. Vector-borne RNA viruses tend to experience less positive selection due to the different defence systems in insect and mammalian hosts, reducing the chances of adaptive mutations favourable for both [57]. Additionally, the small size of RNA viral genomes means that some regions are multifunctional, further limiting the number of tolerable mutations [58,59,60].

Various models have been proposed to estimate the likelihood of new mutants establishing themselves in the environment. One model, proposed by Loverdo, suggests that a mutation is likely beneficial if the survival probability of its neighbours is greater than that of the initial strain [61]. However, this is not a strict requirement, as adaptive mutants can sometimes emerge from more complex evolutionary pathways. The optimal mutation rate in Loverdo’s analysis is that mutation rate which makes the survival of a replicator lineage more likely in the case of an environmental change.

In the case of a high number of non-synonymous mutations, compensatory mutations may be selected, whereas with a low number of compensatory mutations, the site may evolve neutrally (d_N_/d_S_ = 1). High recombination rates can accelerate changes in fitness across different sites in the genome [62].

In the context of spillover, it is important to recognize that a mutant must survive in both the reservoir and the new host. Adaptive mutations beneficial for one host may not be suitable for the other. Maximizing survival of a replicator lineage differs from optimizing adaptation rates (e.g., fixation of adaptive mutants) in a population. For example, if a strain is well-adapted but carries deleterious mutations, the mutation rate that maximizes its survival may be zero, whereas the rate that maximizes adaptation is always positive. For some deterministic models, such as Iranzo’s model, when the replication number is zero, extinction is certain.

Two stochastic models also help describe the impact of mutations on viral evolution:

(1) Eshel’s model: the initial strain cannot survive without mutations; therefore, the optimal mutation rate is strictly positive. In the case ω ≥ (R_2_ − 1)/R_2_ the fit strain will go extinct with certainty, so the optimal mutation rate is bounded below this value (ω = mutation rate; R_1_ = initial strain fitness; R_2_ = reproductive number of the strain) [63];
(2) Alexander and Day’s model, which explores mutation rates in relation to fitness [64].

In the first case, the authors stated that an intermediate level of mutations favoured survival, while in the second case, the authors showed that, despite the existence of an adaptive mutant, mutations can decrease survival if the initial strain is fit enough. Moreover, Loverdo’s analysis describes when mutations are optimal and what factors influence the optimal mutation rates or, more generally, fitness landscapes, considering viral life histories [61].

RNA viruses, despite their high mutation rates, do not always benefit from these mutations. Their rapid adaptation is crucial to avoid extinction in a new host species, but other factors, such as viral load in the reservoir and human exposure, may also drive the emergence of new infections [65].

In stable environments, the mutation rate tends to be low to minimize replication errors [66,67,68,69].

When the environment changes, a few mutants with a high mutation rate can produce adaptive mutations, but these mutants subsequently decline and become extinct when the environment stabilizes [70]. If the mutation rate is slower than the time scale of environmental changes, mutations may be selected to adapt to the new environment as a cost-benefit balance between adaptive mutations and deleterious load. Many studies have explored the evolvability of the mutation rate [71,72,73], but they have not integrated the risk of extinction that follows environmental changes. An essential environmental factor that acts as a driving force is the selective pressure of the host immune system. Viruses have evolved several immune escape strategies that can advantage intra- and inter-host evolution [74].

Another key factor is the duration of infection, which is crucial for transmission. A high mutation rate can decrease the population size and therefore reduce the transmission of new variants to a new host, as it increases the number of pre-existing populations and the emergence of new variants with lower fitness for the new host. Adaptation to the new host involves several replication cycles. Various theories have been proposed to explain this phenomenon. One of them is known as the ‘jack of all trades’ hypothesis [75] or GxE interaction (where G represents the viral genotype and E indicates the environment in which the virus replicates, also known as the host). More recently, this phenomenon has been termed GxGxE interaction, where the phenomenon can be influenced by pleiotropic effects of mutations that occur throughout the genome [76]. However, this is unlikely for small viruses. A study by Lalic on the tobacco mosaic virus shows that fitness variance among different hosts depends on GxE interactions, while 26.13% depends on differences between host species, and only 4.29% on genetic differences between mutants [77]. In the GxE hypothesis, a negative correlation has been observed between the fitness of the primary host and that of the new host. One aspect that needs to be clarified to explain the spillover phenomenon is understanding that antagonist pleiotropy models are useful but overly simplistic, and that more realistic models considering the complexity of host range evolution are needed.

**Complexity of Interaction Between Mutations—**What Does GxE Represent? G represents the point mutation. The virus, once adapted to a new host, shows several mutations compared to its ancestor, which raises the possibility of epistasis. Epistasis, or gene-by-gene (GxG) interaction, indicates that mutations interact with each other rather than simply having additive effects. It is a key factor in adaptive processes as it influences the ruggedness of the adaptive landscape [78,79]. Epistasis can be divided into different types depending on the nature of these interactions. Magnitude epistasis refers to cases where the magnitude of a mutation’s effect depends on the genetic background, while the sign remains constant. Positive magnitude epistasis occurs when the double mutant is fitter than expected from the individual mutations, while negative magnitude epistasis occurs in the opposite case. In contrast, sign epistasis refers to interactions where the genetic background influences not only the magnitude but also the direction (sign) of a mutation’s effect [80]. Another concept, GxGxE, refers to epistatic pleiotropy, where the environment also plays a role.

Many theories assume the host species acts as a constant environment, which is not entirely accurate. Hosts can vary, for instance, through immune system differences or receptor polymorphisms, which may influence viral adaptation. Therefore, host variability, or GxC (where C represents the environment as the host), can also impact spillover events. For example, the Alaska poxvirus infected only one immunocompromised individual, leading to their death [81].

The term “epistasis” was introduced by Bateson to describe how an allele can influence another locus. Later, Fisher redefined epistasis, suggesting that loci can have additive quantitative effects, meaning that multiple genes can influence a single gene, and a gene can be regulated by several others [82,83,84]. Mutations often show pleiotropy, affecting multiple phenotypes simultaneously. Fisher viewed pleiotropy as fundamental to epistasis, which in turn affects fitness [85,86].

**Epistasis** significantly impacts fitness when the effects of mutations deviate from simple multiplicative interactions. It is important to differentiate between unidimensional and multidimensional epistasis [87]. Unidimensional epistasis, also known as directional or mean epistasis, refers to deviations from the linear relationship between mean log fitness and the number of alleles affecting fitness. This form of epistasis can be positive or negative, depending on whether the fitness of genotypes with multiple mutations is higher or lower than expected from independent effects, respectively. Antagonistic epistasis among deleterious mutations and synergistic epistasis among beneficial mutations represent positive epistasis, while the opposite situations indicate negative epistasis. Multidimensional epistasis refers to specific interactions between alleles, offering a more detailed view of the fitness landscape involving these alleles. Additionally, we distinguish between magnitude epistasis, where the combined effect of two alleles deviates from multiplicative effects, without changing their sign. Sign epistasis refers to stronger interaction, where the sign of an allele’s contribution to fitness changes with genetic background [88].

Intragenic epistasis can result from mutations that non-independently affect the stability or activity of an enzyme, while intergenic epistasis may alter protein interactions, disrupting the metabolic network [89].

In adaptive evolution, negative pleiotropy is a prerequisite for “sign” epistasis, as it allows compensatory mutations to offset the negative pleiotropic effects of previous mutations. This also affects genetic drift.

To summarize what has been said so far, epistasis refers to the phenomenon where the effects of one gene (or mutation) are influenced by one or more other genes (or mutations). In viral evolution, this means that certain combinations of mutations can interact in ways that significantly affect a virus’s ability to survive and adapt to new hosts or environments. A recent example that highlights the significance of epistasis in viral transmission is represented by the emergence of SARS-CoV-2 variants, like Delta and Omicron variants, that exhibited mutations which interacted in complex ways, increasing viral resistance to immune responses and enhancing their ability to spread among people [90,91]. Understanding how epistasis affects these mutations can aid in predicting and responding to future variants of the virus.

**Recombination** plays a crucial role in the emergence of new variants, especially among DNA viruses, where it is frequent [92,93]. Although recombination often does not lead to spillover, it helps repair DNA, eliminate mutations, and maintain genome integrity. In RNA viruses, recombination can occur during replication via the copy-choice mechanism, although non-replicative mechanisms also exist [94,95]. While less common in negative-strand RNA viruses, recombination can facilitate new host infections and alter viral tropisms, such as the avian influenza virus that switched from causing respiratory infections in chickens to enteric infections in turkeys through recombination.

From an evolutionary standpoint, recombination helps to maintain fitness when viral populations are composed of variants with low fitness by eliminating deleterious mutations. It also promotes the appearance of divergent genomes able to adapt to new hosts [96].

**Genome reassortment** is another evolutionary force in viruses with segmented genomes, with influenza A being the most well-studied example.

The influenza A virus represents a clear example of how recombination and genome reassortment can lead to the emergence of novel and highly transmissible viral strains. The influenza A virus possesses a segmented genome, which allows genome reassortment (or antigenic shift) when two or more influenza strains infect the same host cell. This reassortment can generate new viral variants with mixed genetic material, a process that has contributed to several influenza pandemics. A prominent example is the 2009 H1N1 influenza pandemic, which emerged as a result of the reassortment of genes from human, swine, and avian influenza viruses. This reassortment produced a virus with novel surface proteins to which the human population had little pre-existing immunity, enabling rapid global transmission [97].

In addition to genome reassortment, antigenic shift, the process by which major changes in viral antigens occur, can lead to the rapid emergence of new strains that evade the immune system. This has also been observed in the 1918 H1N1 Spanish flu and in the 1957 H2N2 Asian flu pandemics.

On the other hand, antigenic drift, a more gradual process, results from the accumulation of point mutations in the genes encoding the hemagglutinin (HA) and neuraminidase (NA) proteins, the primary targets of the immune system. These small genetic changes allow influenza viruses to continuously escape the immune system, leading to seasonal outbreaks and the necessity for annual vaccination.

The evolutionary success of influenza viruses highlights the importance of these mechanisms in shaping viral populations. Antigenic shift, in particular, allows influenza viruses to produce highly divergent genomes, which can spread rapidly in a population and adapt to new hosts, making it of important significance for zoonotic spillover and the emergence of pandemic strains.

Mutations, epistasis, and recombination are key drivers of viral fitness.

Fitness is defined by the effects of individual mutations on viral replication capacity, known as mutational fitness effects (MFEs). Selection acts by favouring variants with beneficial mutations and removing lethal ones. The deterministic forces of natural selection tend to lead the viral population to a maximum fitness, or peak in the fitness landscape, through fixation of adaptive mutations. Protein stability is particularly important in determining MFEs, and mutations that stabilise structural proteins are rapidly removed by negative selection. Viral quasi-species consist of a master sequence and variants that emerge under selective pressures [98].

**Figure 1:**
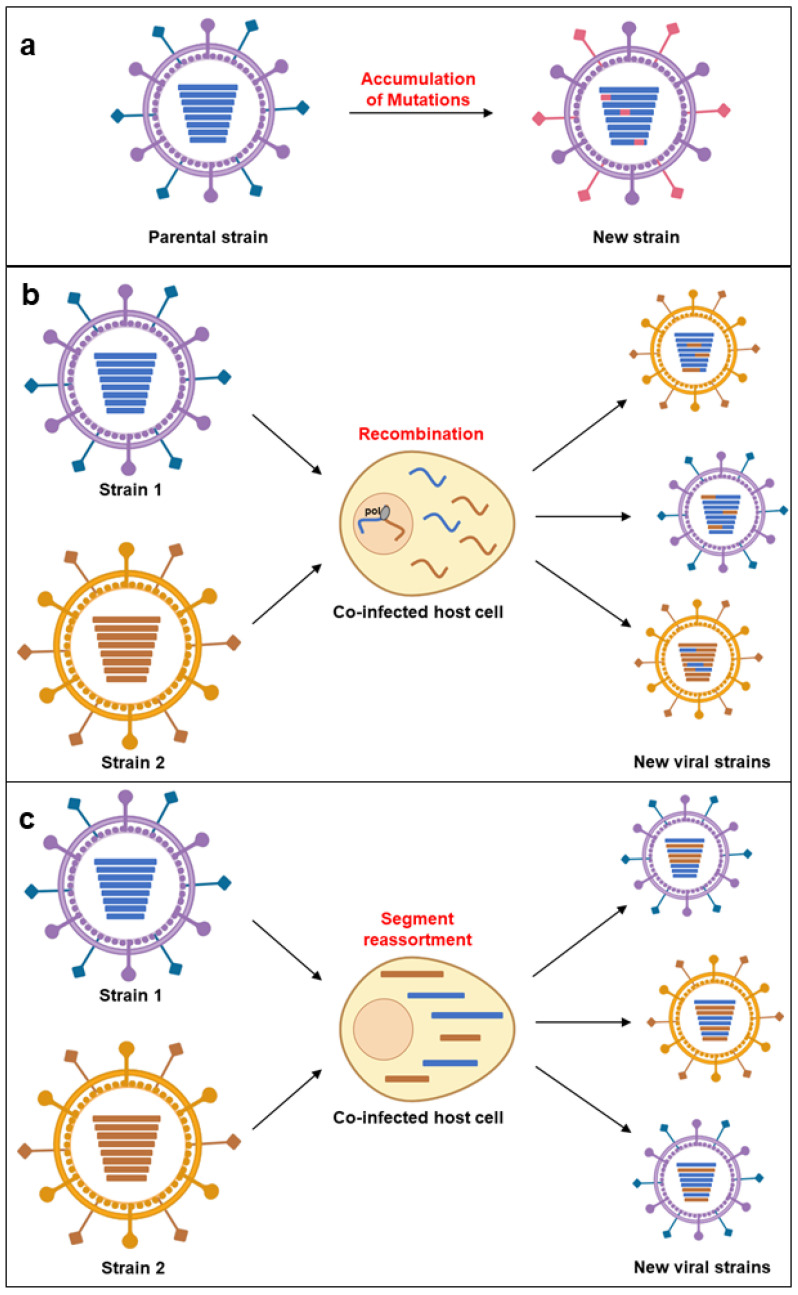
Mechanisms of virus emergence. (**a**) Mutation: random changes in the viral genome can lead to the development of new viral strains with altered properties. (**b**) Recombination: exchange of genetic material between different viruses infecting the same host cell can result in a novel virus. (**c**) Genetic reassortment: in viruses with segmented genomes, the exchange of entire genome segments between co-infecting viruses can generate new combinations of viral genes.

Adaptation is typically measured as an increase in replicative fitness—the ability to produce infectious progeny in a specific environment. Epidemiological fitness describes a virus’s capacity to become dominant over other variants or serotypes [99]. Genetic changes during replication (via mutations, recombination, and segment reassortment) are linked to fitness variation (Figure 1).

In RNA viruses, the wild type is not a fixed sequence but a distribution of sequences. Mutations occur continuously during each replication cycle [96].

Fitness is influenced by both genetic and environmental factors and calculating it can be challenging. Within-host fitness describes a pathogen’s growth within an infected host, while between-host fitness refers to its transmission capacity to a new host. A scale-specific component must be defined. Better models on genome replication and viral packaging can help to obtain a better estimate of the viral mutation rate [100].

Fitness can vary over time and depends on the organism studied. For sexually reproducing organisms, fitness determinants differ from those of asexual beings or viruses. Fitness is also classified as absolute (W, generally ≥ 0), representing the total fitness of a genotype, or relative fitness (w), comparing one genotype’s fitness to the highest fitness observed in the population [101]. The frequency of an allele depends on the relative fitness differences between alleles, not their absolute values.

There are still open questions about fitness, including whether it is determined by single or multiple mutations. Maynard Smith argued that most mutations are rare and thus irrelevant to evolution, while Fisher believed only a small number of mutations drive adaptation [85,102]. The number of mutations contributing to fitness remains uncertain. Theories by Kimura, Ohta, and Gillespie have explored different models, with Gillespie emphasizing the importance of Extreme Value Theory (EVT) in understanding adaptation, particularly in strong-selection-weak-mutation (SSWM) scenarios, where the probability of a beneficial allele being fixed is proportional to its selective advantage [103,104,105,106].

**Anthropocentric factors.** Humans play a significant role in changes that lead to biodiversity loss and precede zoonotic spillover events. Key anthropogenic factors include deforestation, driven by increased urbanization and intensive agriculture, which promotes the loss of medium- and large-sized animals as well as the proliferation of arthropod vectors [107,108]. The drastic decline in predators, essential for controlling populations of small animals, has led to a significant increase in the number of birds and small mammals such as rodents and bats. These species have increasingly moved into urbanized areas, resulting in closer and more frequent contact with humans. The decrease in the number of animal species that could potentially act as alternative hosts to humans in spillover events, combined with the expansion of range and increased population density of reservoir species, has facilitated zoonotic events, particularly involving roboviruses (e.g., Sin Nombre, Hendra, and Nipah viruses) [109,110] and arboviruses (e.g., West Nile, Dengue, Zika, and Chikungunya viruses) [111,112,113]. The zoonotic potential of arboviruses has further escalated due to climate change, which has expanded the range of virus vectors, primarily mosquitoes, into latitudes where they were previously absent. Urbanization and increased human population density also intensify the risk of zoonotic spillover and promote human-to-human transmission, leading to epidemics or pandemics. For instance, measles (MV) emerged from a spillover event of rinderpest morbillivirus (RPV) from cattle to humans over 2500 years ago, with sustained transmission in human populations occurring when cities surpassed 500,000 inhabitants around 300 BCE [114]. In developing countries, growing urban populations near forests have triggered outbreaks of arboviruses and other emerging pathogens, such as Ebola in Sierra Leone.

Additionally, sociocultural factors, such as urban wildlife markets trading diverse species from distant regions, increase the likelihood of interspecies transmission and zoonotic outbreaks, as seen with the 2019 SARS-CoV-2 outbreak in Wuhan, China. The handling and consumption of fresh wild meat, potentially infected with zoonotic pathogens, also poses a significant spillover risk [115].

## 4. Pathogen Adaptation in a New Host

In a new environment, pathogens face selective pressures that can affect their replication capabilities, thus reducing their ability to transmit between individuals of the new host species. These processes determine or influence their capacity for adaptation and, when absent, the virus may become extinct in the new host. Key factors influencing adaptation include competition within the host, population bottlenecks, and the duration of infection. As previously mentioned, RNA viruses exist as a quasi-species—a population of closely related viral variants. However, it remains unclear whether adaptation is primarily driven by selection induced by bottleneck, which leads to the fixation of certain variants, or by evolutionary changes in replication mechanisms that generate variants better adapted in the new host [116,117]. Bottleneck events have been observed, for example, in the transmission of the influenza virus to humans [118]. Variants present at low levels in the donor host—detectable only by next-generation sequencing (NGS)—may be preferentially transmitted to the recipient [119,120,121]. The transmission rate of a virus, however, is not necessarily correlated with its fitness in the donor host [122,123].

A comprehensive theory of evolutionary emergence that accounts for within-host dynamics of competing strains, bottleneck effects, and host-to-host transmission is still needed [124]. Schreiber SJ et al. [125] made an attempt to describe pathogen emergence and adaptation in new hosts using a cross-scale model. Earlier studies on pathogen emergence, such as those by Antia R [126] and Park M [127], focused on single-strain transmission and proposed that adaptation depends on the mutation rate of that strain. In contrast, the quasi-species nature of viral populations suggests that adaptation occurs based on the frequency of variants capable of adapting. The duration of infection plays a critical role in this process; in short-lived infections, there is little time for the virus to escape immune control and adapt to the new host. This has led some researchers to propose the inclusion of the cross-scale reproductive number (α) for mutant virions in addition to the basic reproductive number (R_0_). For a mutant virus to establish itself, each transmitted mutant virion must, on average, produce one or more mutant offspring (α > 1). If α < 1, adaptive variants decrease, and the virus fails to spread in the new host population [128]. This phenomenon would explain how the virus population can undergo the growth required to overcome the spillover threshold.

**Figure 2:**
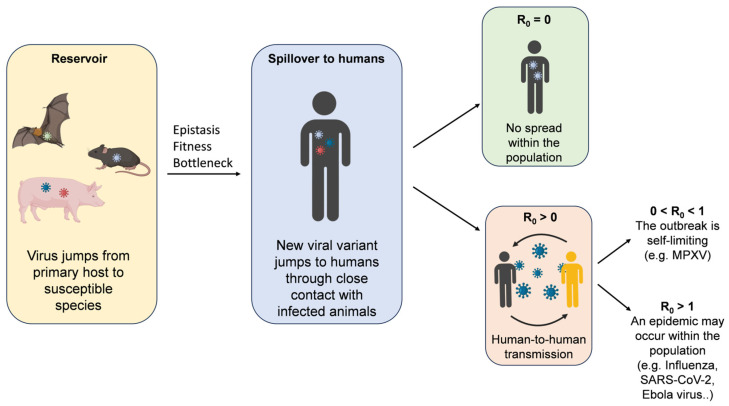
Pathogen spillover events. Each viral strain is represented by a different colour.

The bottleneck phenomenon is also believed to reduce the fitness of certain strains during host jumps, which is why multiple replication cycles are essential to acquire fitness in a new host. As replication progresses, some mutations may become fixed in the new host, while others do not. Once the pathogen is established in the new host species, its spread is determined by R_0_, the number of secondary cases generated by a single case [129]. When R_0_ = 0, there is no spread within the population. If 0 < R_0_ < 1, the outbreak is self-limiting, whereas when R_0_ > 1, an epidemic may occur within the population [130]. The effective reproduction number (R_eff_) can also be used, indicating the average number of cases in a population that includes both susceptible and immune individuals (e.g., due to vaccination) [131,132]. R_0_ is equal to 0 for viruses such as West Nile or rabies. When 0 < R_0_ < 1, as seen with Monkeypox or Nipah virus, the outbreak remains self-limiting. For viruses such as influenza, SARS-CoV-2, and Ebola, R_0_ > 1 [133] (Figure 2).

Bottlenecks significantly influence both inter-host and intra-host transmission by intensifying genetic drift relative to selection pressures. This can move viral populations into new selective regimes, such as during host shifts or immune escape events [74,134,135].

Several factors can influence viral ability to spread after adapting to a new host. For example, some viruses can be transmitted via extracellular vesicles, as seen in hepatitis A virus [136], enteroviruses, Marseilleviruses [137], noroviruses, and rotaviruses [138]. Another important phenomenon is polyploidy, where multiple genomic copies exist within the same virion, as observed in Ebolavirus [139]. Viral transmission can occur through cellular specific structures such as plasmodesmata, immunological or neurological synapses, nanotubes, or syncytia, facilitating the spread of viral genomes between cells. These mechanisms are all examples of collective spread, where multiple virions or genomic materials are transmitted together, enhancing viral fitness and transmission efficiency.

Although the collective spread of viruses is well-documented, its implications for viral fitness, diversity, and evolution are not yet fully understood. High multiplicity of infection (MOI) could enhance viral fitness through genetic complementation between co-infecting mutants, which is particularly important for RNA viruses with high mutation rates [140,141,142]. Complementation is considered particularly important in RNA viruses, which, due to their high mutation rates, easily accumulate deleterious mutations [143,144]. Complementation, which can influence mutation rates and therefore fitness, can occur in various systems of collective viral spread, including extracellular enterovirus vesicles [145] and poliovirus aggregates [146]. Many theories and models that calculate viral fitness do not consider collective viral spread. Moreover, high MOI promotes the evolution of defective interfering particles (DIPs) and other defective virus types that replicate at the expense of fully functional ‘helper’ viruses [147,148]. Since DIPs have shorter sequences and benefit from helper viruses without reciprocity, they can take over the population at high MOIs, dramatically reducing viral population fitness [149,150]. On the other hand, the occurrence of DIPs may be a valuable evolutionary mechanism to side-track the immune system by allowing the infection of wild-type virus. Hence, although there are benefits derived from complementation, these can be weakened because they promote the emergence of DIPs. According to the model developed by Segredo-Otero [151], when mutations are rare, genetic complementation has no effect on the average population fitness, whereas at high mutation rates, complementation could drive an error catastrophe. An error catastrophe is a situation in which selection fails to maintain non-mutated sequences at a higher frequency than low-fitness mutated sequences [152,153]. The authors noted that although complementation does not have positive effects on population fitness at equilibrium, it nonetheless increases robustness against deleterious mutations and transiently improves mean fitness [151].

Various external factors to viruses influence spillover, such as proximity between reservoir and recipient species, as well as viral survival in vectors (in the case of arboviruses) [154]. An example of adaptation and survival in a new vector is represented by chikungunya virus, where an alanine-to-valine substitution at position 226 of the E1 glycoprotein allowed it to make the vector jump within the mosquito genus *Aedes*, switching from *A. aegipty* to *A. albopictus* [155].

These external factors are also exemplified in notable cases such as the Nipah and Hendra viruses. The Nipah virus, first identified in Malaysia in 1998, is believed to have spilled over from fruit bats to pigs and subsequently to humans. This event was facilitated by environmental factors such as the close proximity of human populations to bat habitats and intensive pig farming practices, which created conditions for interspecies transmission. Genetic analyses showed that the Nipah virus adapted to both pig and human hosts, resulting in severe outbreaks with high mortality rates. Similarly, the Hendra virus, which emerged in Australia in 1994, was transmitted from flying foxes (fruit bats) to horses and then to humans. In this case, the spillover was influenced by environmental changes that disrupted bat habitats, increasing contact between bats and domestic animals. The Hendra virus demonstrated a remarkable ability to adapt to its new hosts, highlighting the critical role of genetic variability and ecological conditions in spillover dynamics [156]. These case studies underscore the importance of understanding the factors that contribute to spillover events, as they can inform predictive models and prevention strategies for EIDs.

The number of infected individuals in the reservoir and the intensity of infection are also crucial for spillover, as larger reservoir populations can support greater genetic diversity, increasing the likelihood of transmission to a new host [13].

Seasonal variations in reservoir populations, such as those of *Mastomys natalensis* (Lassa virus) and *Peromyscus maniculatus* (Sin Nombre virus), can lead to genetic bottlenecks in both the pathogen and the host, affecting the timing and probability of emergence. Deterministic and stochastic models can be used to assess the influence of demographic and epidemiological factors on pathogen diversity and emergence risk, with simulations showing that reservoir size and infection levels strongly influence spillover dynamics.

In addition, the ability of eukaryotic cells to detect viral genomes with high GC content, via enzymes like the zinc finger antiviral protein (ZAP), presents another barrier to adaptation and transmission in new hosts [157,158]. Most viruses have an A-rich, C-poor genome composition, resembling the genomes of insects, birds, and mammals, which may help them evade detection by ZAP.

**Figure 3:**
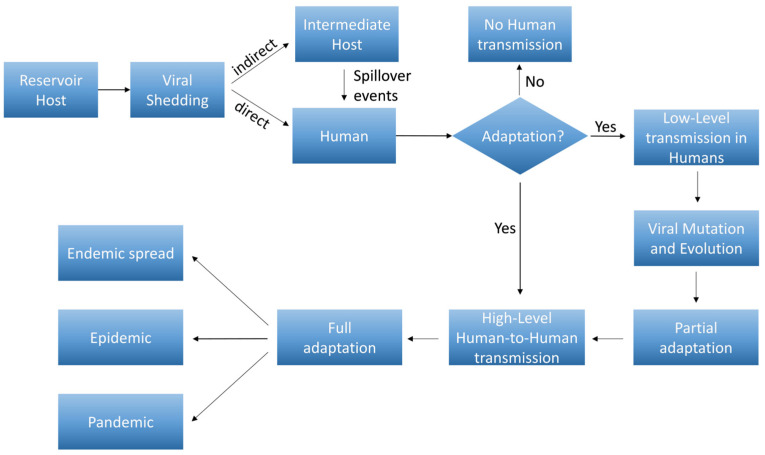
Flowchart of different stages of viral adaptation from reservoirs to humans. Reservoir Host: the virus resides in a natural host (e.g., bats, rodents) where it is well-adapted, causing minimal or no disease in the host. Viral Shedding: the virus is shed from the reservoir host, often through bodily fluids (e.g., saliva, urine, feces), potentially contaminating the environment or being transmitted to an intermediate host (spillover events) or directly to humans. Human Infection: initial infections may be sporadic and may not efficiently spread between humans (no adaptation). The virus adapts enough to allow limited transmission between humans. Viral Mutation and Evolution: the virus accumulates mutations that enhance its ability to infect and replicate in humans, leading to a partial adaptation. High-Level Human-to-Human transmission: the virus becomes better adapted to the human host, allowing sustained human-to-human transmission, often through respiratory droplets, bodily fluids, or other routes. Full adaptation: at this stage, the virus may spread rapidly within human populations, leading to endemic outbreaks, epidemic or pandemics. Over time, the virus may continue to mutate and adapt, potentially becoming more or less virulent as it stabilizes within the human population.

The major stages of viral adaptation from reservoirs to human are schematically represented in Figure 3.

## 5. Discussion

Spillover as well as zoonosis, when the host is human, are events that depend on viral growth rate, expansion, or host-adaptation that are related and are dependent on a number of variables, many of which are challenging to predict with precision. A few crucial factors have been determined; these include the reservoir species’ death rate and the new host’s level of immunological tolerance, both of which have the potential to function as bottlenecks. However, there is still much to learn about adaptation to a new host, so more research is necessary. Despite significant advances in our understanding of viral dynamics, further investigations are needed to identify specific factors that can influence spillover, particularly regarding the factors that influence viral fitness, adaptation, and transmission in new hosts.

In addition to the biological factors influencing viral adaptation, recent technological advances have significantly enhanced our ability to study viral genetic variability within reservoirs and track the emergence of new variants. Next-generation sequencing (NGS) platforms such as Illumina and Oxford Nanopore now allow for rapid and precise sequencing of entire viral genomes, facilitating the identification of mutations at different frequencies within viral populations [159,160]. These technologies provide critical insights into the dynamics of viral evolution, especially during zoonotic spillover events. Furthermore, CRISPR-based genome editing has revolutionized the study of viral genetics, enabling precise manipulation of viral genomes to explore the functional consequences of specific mutations [161]. Despite these advancements, challenges remain in detecting low-frequency variants, and access to these technologies is still limited in low-resource settings, emphasizing the need for more affordable tools for global viral surveillance.

Current mathematical models often begin with assumptions that oversimplify the complex reality of viral emergence. For example, they may assume a constant virus growth rate in both the reservoir and the new host, which fails to capture the dynamic nature of viral transmission and adaptation. Due to these limitations, we have chosen not to investigate the specifics of these models in this review, leaving such discussions to specialists in mathematical biology. Instead, we have focused on the biological and ecological factors that drive the emergence of new viral variants capable of causing spillover events. We emphasize the critical steps that facilitate the adaptation of these variants in both the reservoir and the new host, with a particular focus on phenomena such as epistasis and bottlenecks that influence the establishment and fixation of beneficial mutations.

One of the main challenges in understanding viral emergence is the unpredictability of the evolutionary processes that drive it. The role of bottlenecks in selecting specific variants for transmission is still debated. Bottlenecks can reduce viral diversity, but they may also allow low-frequency variants that are better adapted in the new host, becoming predominant. Moreover, the ability of a virus to adapt depends on several factors, including competition within the host, the duration of infection, and the fitness of specific strains during transmission.

Lethality and transmissibility are influenced by key epidemiological parameters such as the reproductive number (R_0_) and the incubation period between infection and symptom onset. R_0_, in particular, is crucial for understanding how a virus spreads in a population. When R_0_ is greater than 1, the pathogen has the potential to cause an epidemic, as seen in cases like SARS-CoV-2, Ebola, and influenza. However, accurately estimating these parameters is challenging, particularly in the early stages of a viral emergence. The descriptions of spillover phenomena provided in this review illustrate the many gaps in our ability to predict the emergence of new viruses capable of infecting humans or other novel hosts.

In addition to these biological and epidemiological factors, the mode of transmission plays a significant role in shaping viral spread and adaptation.

Another critical factor is the role of host immune defences, particularly the ability of host cells to detect and eliminate viral genomes with high GC content. This defense mechanism highlights the challenges viruses face in adapting to new hosts, as they must evade both innate and adaptive immune responses to establish successful infections [157,158].

Environmental and ecological factors are key determinants of spillover risk, such as the proximity between reservoir species and potential new hosts, and the prevalence of infection within the reservoir population. Seasonal variations in reservoir populations, such as those observed in rodent species infected with hantaviruses, can induce genetic bottlenecks in both the reservoir and the virus, affecting the timing and probability of emergence. Climate change could expand the distribution range of species of potential viral vectors and reservoirs, broadening the geographical areas of distribution and epidemiology of viruses. Deforestation and permafrost reduction could bring out new potential zoonotic viruses currently confined to their reservoir species or frozen in ice. Furthermore, in particular geographical areas of the world, the close cohabitation of humans, domestic and wild animal species, as well as the presence of wet markets have established the conditions for the outbreak of zoonotic viruses and pose a worldwide risk to human health.

In summary, the emergence of a new virus capable of infecting a novel host is a complex interplay of factors including viral evolution, host-pathogen interactions, and ecological conditions.

There are still many gaps preventing the prediction of the emergence of a new virus capable of adapting to humans or a new host; therefore, estimating a reliable model rate remains challenging [162,163].

In addition to efforts to find new antiviral drugs and vaccines, there is an urgent need to implement surveillance systems and best practices when it comes to ecological virus containment to help contain epidemics and pandemics. The COVID-19 pandemic has taught us that it is crucial to enhance our understanding of the factors triggering zoonotic spillover. This knowledge is essential for governments and health organizations worldwide to implement better actions to control and prevent the emergence of new epidemics and pandemics. International strategies to prevent another global health emergency include both ecological measures (reducing deforestation and defaunation, increasing biodiversity, permanently closing wet markets, combating wildlife trafficking, reducing urbanization in forest areas, and vector control through disinfestation) and health and research efforts (enhancing veterinary health surveillance, improving healthcare infrastructure, and increasing investments in identifying and studying new pathogenic viruses to develop novel preventive and therapeutic strategies). In conclusion, by focusing both on preventing zoonotic spillovers and advancing new intervention strategies (prevention and treatment), we can significantly reduce the loss of human life and healthcare costs associated with the emergence or re-emergence of pathogenic viruses.

## Data Availability

The original contributions presented in the study are included in the article, further inquiries can be directed to the corresponding author.

## Glossary

Antagonistic Pleiotropy: Occurs when a single mutation or gene affects multiple traits in opposite ways, enhancing one trait while potentially compromising another. In the context of viral evolution, it often refers to trade-offs between adaptation to new hosts and fitness in the original host. Antigenic Drift: The gradual accumulation of mutations in viral surface proteins, leading to small changes that allow the virus to evade immune responses, often seen in seasonal influenza viruses. Antigenic Shift: A genetic change caused by the reassortment of viral genes, leading to significant changes in antigens that can result in pandemics due to the lack of immunity in the population. Bottleneck Effect: A phenomenon in which a virus passes through a small population size, often during host transmission, resulting in reduced genetic diversity. This can intensify genetic drift, affecting viral adaptability. d_S_/d_N_ Ratio: A measure of the selection pressure on protein-coding genes by comparing the rate of synonymous mutations (d_S_), which do not affect the protein, to nonsynonymous mutations (d_N_), which do. A ratio > 1 indicates positive selection, while a ratio < 1 implies purifying selection. Epistasis: Refers to interactions between genes where the effect of one gene is modified by one or more other genes. This interaction influences the fitness of organisms, especially in viral evolution, where it shapes the fitness landscape by altering the effects of mutations. Error Catastrophe: A situation in which the accumulation of mutations in a viral population exceeds a critical threshold, leading to a loss of functional viral particles and potential population collapse. Fitness Landscape: A conceptual representation of the relationship between genotypes and their reproductive success. In viral evolution, the landscape shows how mutations alter fitness, with peaks representing high fitness and valleys representing low fitness genotypes. Mutation Rate: The frequency at which changes occur in the genome. RNA viruses have notably high mutation rates, enhancing their ability to adapt to new hosts but also increasing the likelihood of deleterious mutations. Pleiotropy: The phenomenon where one gene influences multiple traits. In viral genomes, this can result in viral adaptation and fitness across different hosts. Positive Selection: A process where beneficial mutations are favoured and become more common in a population. In the context of viral evolution, this often refers to mutations that enhance viral fitness, facilitating transmission and survival. Quasispecies: A group of genetically related viruses within a population that have slight mutations. This diversity provides adaptability to new environments or hosts, particularly important for RNA viruses with high mutation rates. Reassortment: A process where segmented viruses, such as influenza, exchange genetic material when co-infecting the same host cell, leading to new viral strains with mixed characteristics, potentially allowing spillover events. Recombination: The exchange of genetic material between viruses during co-infection, leading to the creation of novel viral genotypes. Reproductive Number (R_0_): A measure of how contagious a pathogen is, representing the average number of secondary infections produced by a single infected individual in a fully susceptible population. When R_0_ > 1, an epidemic is likely. Sign Epistasis: A type of gene interaction where the effect of a mutation on fitness depends on the genetic background. For instance, a mutation might be beneficial in one context but deleterious in another.
